# Study on Surface Discharge Characteristics of GO-Doped Epoxy Resin–LN_2_ Composite Insulation

**DOI:** 10.3390/polym14071432

**Published:** 2022-03-31

**Authors:** Yunqi Xing, Yuanyuan Chen, Ruiyi Yuan, Zhuoran Yang, Tianyi Yao, Jiehua Li, Wenbo Zhu, Xiaoxue Wang

**Affiliations:** 1 State Key Laboratory of Reliability and Intelligence of Electrical Equipment, Hebei University of Technology, Tianjin 300401, China; yqxing@hebut.edu.cn (Y.X.); yychebut@163.com (Y.C.); 2 Key Laboratory of Electromagnetic Field and Electrical Apparatus Reliability of Hebei Province, Hebei University of Technology, Tianjin 300401, China; 3 Chengdu Power Supply Company, State Grid Sichuan Electric Power Company, Chengdu 610041, China; yrysgcc_cd@163.com; 4 Nanjing Power Supply Company, State Grid Jiangsu Electric Power Company, Nanjing 210019, China; yangzhuoran@126.com (Z.Y.); 13515100302@139.com (T.Y.); 5 State Grid Tianjin High Voltage Company, Tianjin 300143, China; w344132814@126.com; 6 China Southern Power Grid Research Institute Co., Ltd., Guangzhou 510080, China; zhuwb@csg.cn

**Keywords:** HTS energy pipeline, epoxy resin, LN_2_, partial discharge, surface flashover

## Abstract

Superconducting power lead equipment for epoxy insulation, such as high-temperature superconducting DC power or liquefied natural gas energy pipelines, as well as high-temperature superconducting cables, has long been used in extreme environments, from liquid nitrogen temperatures to normal temperatures. It is easy to induce surface discharge and flashover under the action of strong electric field, which accelerates the insulation failure of current leads. In this paper, two-dimensional nano-material GO was used to control the electrical properties of epoxy resins. The DC surface discharge and flashover characteristics of the prepared epoxy resin–GO composite insulation materials were tested at room temperature with liquid nitrogen. The surface discharge mechanism of the epoxy resin–GO composite insulation materials was analyzed. The experimental results show that the insulation properties of epoxy composites doped with GO changed. Among them, the surface flashover voltage of 0.05 wt% material is the best, which can inhibit the discharge phenomenon and improve its insulation properties in extreme environments, from room temperature to liquid nitrogen temperature. It is found that the development process of surface discharge of composite insulating materials under liquid nitrogen is quite different from that under room temperature. Before critical flashover, the repetition rate and amplitude of surface discharge remain at a low level until critical flashover. Furthermore, the voltage of the first flashover is significantly higher than that of the subsequent flashover under the action of the desorption gas on the surface of the composite insulating material and the gasification layer produced by the discharge. Given that the surface flashover voltage of 0.05 wt% epoxy composite is the best, the research and analysis of 0.05 wt% composite is emphasized. In the future design of superconducting power lead insulation, the modification method of adding GO to epoxy resin can be considered in order to improve its insulation performance.

## 1. Introduction

In order to achieve the goal of “carbon peak and carbon neutralization”, it is urgent for power energy system to reduce transmission and distribution losses, vigorously develop clean energy, and enhance the proportion of new energy generation in the future development [1,2]. Affected by the highly unbalanced distribution of energy resources and load centers in China, the basic pattern of “west-to-east power transmission” and “west-to-east gas transmission” will be maintained for a long time [3]. Superconducting transmission technology can reduce transmission loss by 80% in DC transmission system due to its advantages of no resistance loss, large capacity, and small size, which can greatly improve transmission efficiency, stability, and capacity of power grid [4,5]. However, the cooling medium required to maintain the operation of the superconducting transmission system accounts for a large proportion of the total loss of the transmission system, while the superconducting DC power or liquefied natural gas (LNG) energy pipeline using LNG at 77–110 K as a cooling medium can maintain the superconducting operation of copper oxide superconductor, realize long-distance and low-loss power transportation, and realize liquefied collection and transportation of clean energy. It provides a better option for long-distance transmission of power and clean energy [6,7,8]. In the superconducting DC energy pipeline, according to different design schemes, the superconducting cable insulation is in a low-temperature working environment of 77–110 K. For example, the operating temperature range of ±100 kV/1 kA in a superconducting power LNG hybrid energy pipeline developed by China Electric Power Research Institute is 85–90 K [6]. The mechanical properties of epoxy composite insulation materials commonly used for current lead insulation will be significantly reduced in extreme low-temperature environment. Partial cracks and other defects are prone to occur under the coupling action of electric and thermal stress, which induces partial discharge and accelerates insulation failure [9,10,11,12].

W. G. Li et al. studied and analyzed the flashover voltage characteristics of epoxy resin. It was found that under the condition of liquid nitrogen (LN_2_), the flashover causes the surface of epoxy resin to show electro-traces due to carbonization, and generates a LN_2_ gasification layer which is adsorbed on the surface of flashover channel. The channel shape of multi-flashover along the surface is highly similar, while the repeated flashover voltage value is significantly reduced and the discharge delay is reduced [13]. S. T. Dai et al. found that the relationship between the flashover voltage along the surface and the size of the flashover gap of G10 epoxy material under LN_2_ condition has a saturation tendency, and it is difficult to improve the insulation performance by increasing the insulation size [14]. Operating conditions in the LN_2_ temperature zone put forward higher requirements on the performance of epoxy resin. In order to improve the insulation reliability of epoxy resin, scholars around the world attempt to adopt a nano-doping modification method in order to improve the surface discharge performance of epoxy resin in extreme low-temperature environments [15,16,17]. Y. Zeng found that the thermal expansion coefficient of composites can be reduced [18] by doping nanoparticles with low thermal expansion coefficient in the epoxy composite insulating material, while the insulating material with low thermal expansion coefficient can cooperate better with the conductor and reduce the generation of crack defects. Y. J. Lee et al. used nano-alumina and titanium dioxide to regulate the electrical properties of epoxy resin and found that nano-alumina increased the AC breakdown voltage of epoxy resin composites more [19] under LN_2_ conditions. However, because the surface of nano-particle filler is quite different from that of epoxy resin matrix, agglomeration is easy to occur during the preparation process, which will result in a decrease in the breakdown resistance of composites. J. H. Koo et al. found that modification of nano-filler under LN_2_ did not significantly increase the AC/DC interfacial breakdown voltage of epoxy nanocomposites [20]. 

In conclusion, there are some differences in the modification effect of epoxy composite insulating material in the performance study of epoxy composite insulating material under low-temperature environments. Furthermore, the modification process and method need to be studied in further detail. The properties of insulating materials vary after doping different proportions of nano materials, but it is undeniable that nano doping modification can effectively regulate the insulating properties of composites. GO has excellent electrical and thermodynamic properties. It is not only an ideal isotropic thermal conductive material and the best conductive material at room temperature, but also has a high specific surface area. Most importantly, the active oxygen-containing groups on the surface of GO enable remarkable results at low doping levels. Graphene is considered as an ideal filler for improving electrical and thermal conductivity of resin matrix composites. Therefore, nano GO with a higher specific surface area was used to dope and modify bisphenol F-type epoxy resin to insulate a superconducting DC energy pipeline, which can effectively regulate the electrical properties of epoxy resin at a very low doping ratio. The conductivity, dielectric constant, and trap distribution of epoxy resin and its composites were measured. Then, the development of surface discharge and flashover process under LN_2_ was studied, and compared with room temperature conditions. Finally, the mechanism of surface discharge under LN_2_ was discussed. Thus, a reference for the development of superconducting DC energy pipeline insulation is provided.

## 2. Preparation and Experimental Method of Epoxy Resin Composite Material

### 2.1. Preparation of Epoxy Resin–GO Composite

(1) Weigh an appropriate amount of bisphenol F epoxy resin into a round bottom flask and place it in a 60 °C water bath to increase the fluidity of the epoxy resin.
(2) Add GO aqueous dispersion into epoxy resin, stir at high speed for 1 h (60 °C), and then conduct ultrasonic treatment for 1 h to increase the dispersion of GO in epoxy resin.
(3) Set the water bath temperature at 60 °C, vacuumize the mixed liquid, and treat the mixed liquid with water for 2 h to remove the water in the mixed liquid.
(4) Add the DETDA curing agent according to the mass ratio of curing agent to epoxy resin of 1:4, stir the mixture at a high speed in a 20 °C water bath, and fully degas it for 1 h.
(5) Pour the mixture into the polytetrafluoroethylene mold, put it into the drying oven, and then pre-solidify it at 80 °C for 5 h and at 150 °C for 5 h.

**Figure 1:**
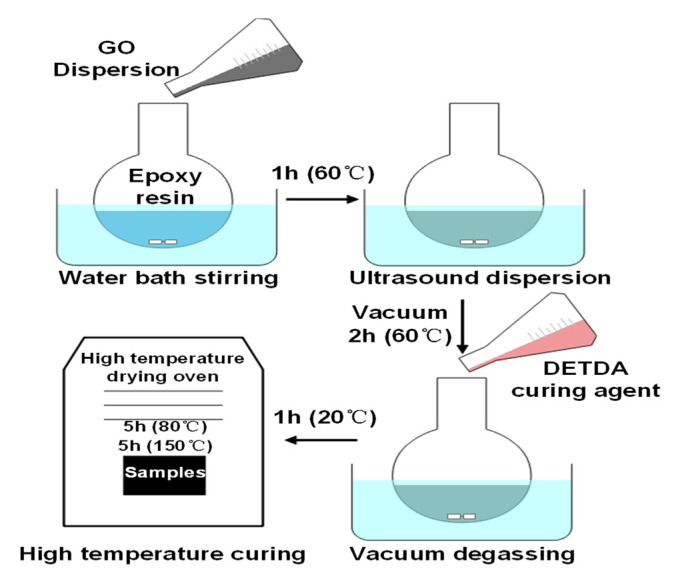
Preparation process of epoxy resin–GO nanocomposites.

The preparation process is shown in Figure 1.

The epoxy resin-type GY285 bisphenol F (DGEBF) and the amine curing agent diethyltoluene diamine (DETDA), produced by the Huntsman Company, were used for sample preparation. Due to the large amount of functional groups, such as the hydroxyl group, the carboxyl group, and the epoxy group on the surface of nano GO, good compatibility with the epoxy matrix was achieved, and a single-layer two-dimensional nanostructure with a positive specific surface (up to 2630 m^2^/g) was attained. A large number of interfacial areas can be introduced by doping a small amount of nano GO to control the trapping characteristics of composites. Therefore, GO was selected as a doping modification material in this paper. The GO used was produced by Hunan Fenghua Material Co., Ltd., and its transverse dimension was 5.7 um, its single layer rate was 92.5%, and its oxygen index was 48.5. In this paper, 0 wt%, 0.02 wt%, 0.05 wt%, 0.1 wt%, and 0.2 wt% GO epoxy composites were prepared. GO material can achieve good dispersion and stability in epoxy resin material without modification, thus improving the electrical properties and toughness of composites.

### 2.2. Surface Discharge Test Method for Epoxy Resin–GO Composites

**Figure 2:**
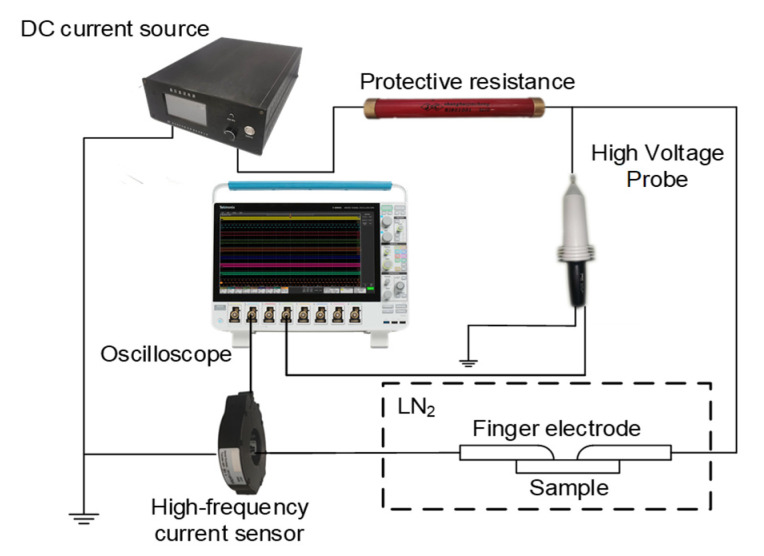
DC surface discharge and flashover voltage test device.

The dielectric constant, DC conductivity, and surface potential decay of epoxy nanocomposites were measured by wide-band dielectric spectroscopy, the three-electrode method, and the surface potential method [21,22,23]. The initiation and development process of partial discharge and flashover voltage of composite materials were measured by a DC flashover test device, as shown in Figure 2. The DC flashover experimental device used a positive DC voltage source with an output amplitude of 0~50 kV, and was connected to the high voltage pole of the finger electrode with a current limiting resistance of 2 MΩ. Pulsing current signals generated by partial discharge were measured by passing a high-frequency current sensor through the ground wire. Voltage values during flashover development were measured by a high-voltage probe connected in parallel at both ends of the finger-type electrode, and voltage signals were transmitted to the oscilloscope via a coaxial cable to record the flashover voltage.

## 3. Dielectric Properties and Surface Discharge Process of Epoxy Resin–GO Composites

### 3.1. Dielectric Properties of Epoxy Resin–GO Composites

**Figure 3:**
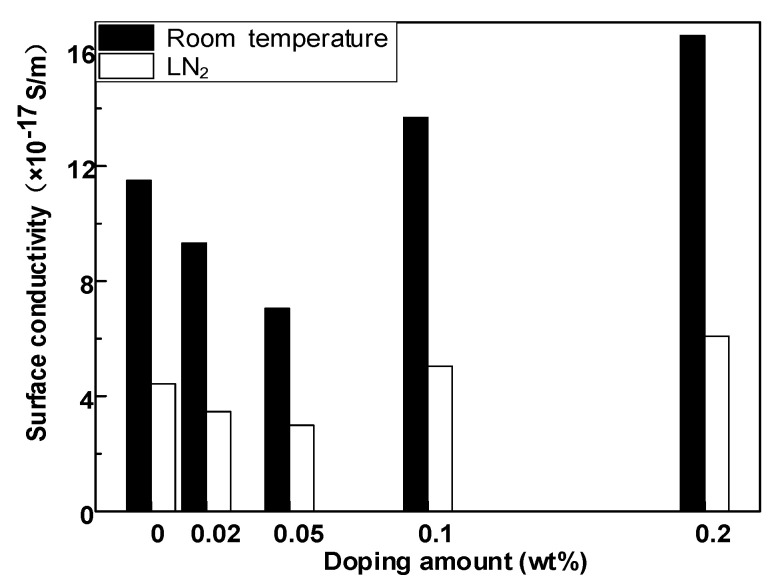
Surface conductivity of epoxy resin–GO composites.

Figure 3 shows the DC surface conductivity of composites with various doped mass fractions at room and LN_2_ temperatures. Under the condition of LN_2_, the surface conductivity of epoxy resin without modification was 4.43 × 10^−17^ S/m. Following an increase in the GO doping mass ratio, the surface conductivity of composites first decreased and then increased. The surface conductivity of the composites reached the lowest when the nano GO doping ratio was 0.05 wt%, i.e., 2.99 × 10^−17^ S/m. The surface conductivity of the composite material was higher than that of the pure epoxy resin when the doping ratio exceeded 0.1 wt%, and the surface conductivity of the composite material reached 6.08 × 10^−17^ S/m at 0.2 wt%. The changing trend of surface conductivity at room temperature was the same as that under LN_2_, but the surface conductivity at room temperature was significantly higher than that under LN_2_.

Due to the inhibited thermal movement of molecular structure of epoxy composite insulating material at low temperature, the conductivity of polymer material was approximately related to temperature, as follows:

(1) $$\gamma \left(T,E\right)=3.2781\exp\left(\frac{-0.56q}{kT}\right)\times \frac{\sinh\left(2.7756\times 10^{-7}\left|E_{Total}\right|\right)}{\left|E_{Total}\right|}$$

The parameters in Equation (1) are the Boltzmann constant k, the thermodynamic temperature T of insulating material, and the unit charge Q. The thermal movement of molecular structure in the epoxy composite insulating material was inhibited in the LN_2_ temperature zone, while the carrier transfer and charge separation from hydrazine were more difficult, which decreased the surface conductivity of the material. The correlation between surface conductivity and doping ratio of epoxy resin–GO composites at LN_2_ temperature was weaker than that at room temperature.

**Figure 4:**
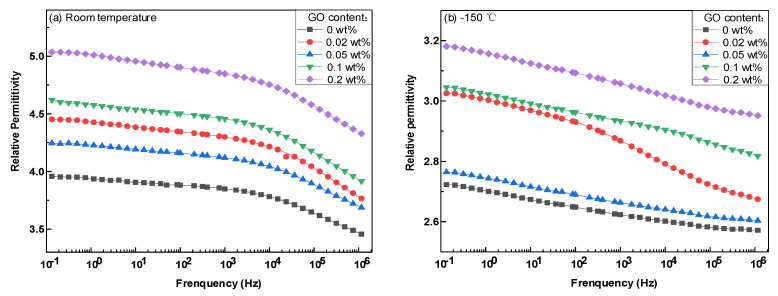
Spectrum of relative permittivity of epoxy nanocomposites at room temperature and −150 °C. (**a**) Spectrum of relative permittivity of epoxy nanocomposites at room temperature; (**b**) Spectrum of relative permittivity of epoxy nanocomposites at −150 °C.

Figure 4a,b show spectrograms of a relative dielectric constant of composites with different doped mass fractions at room temperature and −150 °C, respectively. With an increase in the doping amount of nano GO, due to the sheet structure of two-dimensional nano GO, its surface energy was higher, and the overlapping interface area could occur when the doping amount was low, while the dielectric constant of the composite material increased rapidly due to the presence of a large number of interlayer polarized charges in the interface area. At the doping ratio of 0.05 wt%, the polarization process of the molecular chain was inhibited and the dielectric constant of the composites decreased due to the existence of a large number of non-overlapping interfacial areas in the composites. At −150 °C (near power frequency and 120 K temperature), the movement of molecular chains and polar groups in the epoxy composite insulating material was inhibited, which extended the relaxation time of dipole polarization, making it difficult to match the change frequency of the electric field, resulting in a decrease in the overall dielectric constant of the composite material, while the overall change trend was basically the same.

### 3.2. Surface Discharge Process and Flashover Characteristics of Epoxy Resin–GO Composites

**Figure 5:**
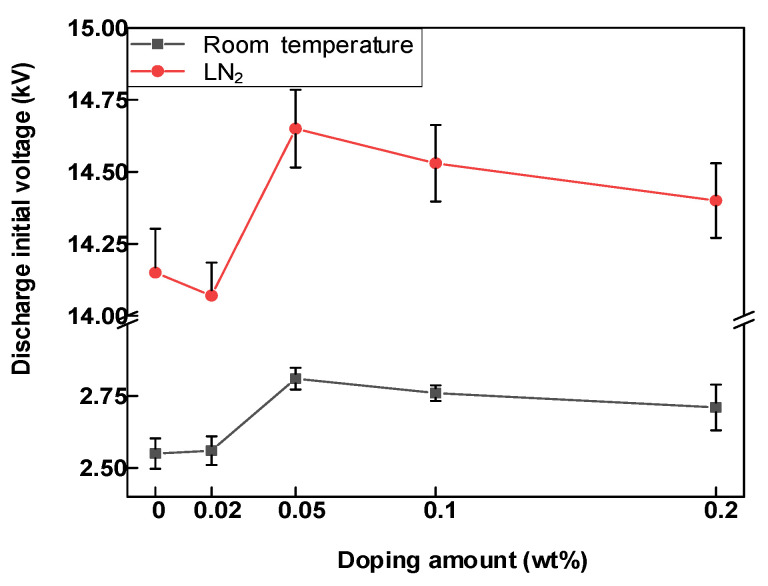
DC surface discharge initial voltage of epoxy resin–GO composites.

**Figure 6:**
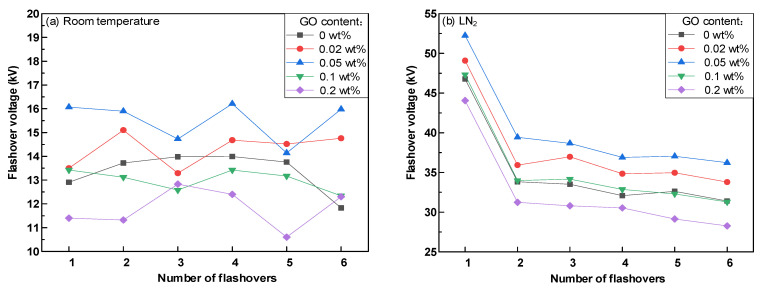
Variation characteristics of discharge repetition rate. (**a**) Variation characteristics of discharge repetition rate at room temperature; (**b**) Variation characteristics of discharge repetition rate at LN_2_.

Figure 5 shows the variation of the onset voltage of surface discharge of epoxy resin–GO composites at room and LN_2_ temperatures. With an increase in the doping mass ratio, the onset voltage of surface discharge increased, but only slightly. The starting discharge voltage of the epoxy resin–GO composite with a 0.05% wt doping ratio was the highest. The epoxy resin–GO composite with 0.05 wt% arguably had the best effect of enhancing flashover voltage and suppressing discharge. Figure 6a,b show the regularity diagrams of flashover voltage change with flashover times of samples with different GO doping mass fractions at room temperature and LN_2_ temperature, respectively. No obvious relationship between the flashover voltage and the number of flashovers at room temperature was detected. However, the first surface flashover voltage of all epoxy composites under LN_2_ conditions was significantly higher than that of subsequent flashover. The flashover voltage decreased, and gained stability as the number of tests increased during subsequent flashover voltage tests. Under room temperature and LN_2_ conditions, the flashover voltage of 0.05 wt% epoxy resin–GO composite material was higher than that of other materials. Combined with the initial discharge voltage value in Figure 5, 0.05 wt% epoxy composite material arguably had the best suppression effect on the discharge signal. Thus, in this paper, 0.05 wt% epoxy composite material was mainly used for test and data analysis. It can be seen from Figure 5 and Figure 6 that the discharge starting voltage at a normal temperature of epoxy composites with any doping ratio was higher than that at LN_2_, while the discharge starting voltage at LN_2_ was much higher than that at room temperature for the surface flashover voltage.

**Figure 7:**
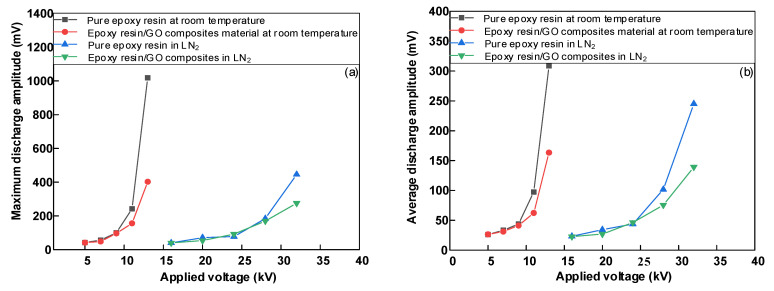
Surface discharge amplitude of epoxy resin–GO composites. (**a**) Surface discharge amplitude of epoxy resin–GO composites at room temperature; (**b**) Surface discharge amplitude of epoxy resin–GO composites at LN_2_.

Figure 7a,b show the variation of maximum and average discharge amplitudes of pure epoxy resin and 0.05 wt% doped epoxy resin–GO composites at room and LN_2_ temperatures, respectively. Figure 7 shows that the maximum discharge amplitude and average discharge amplitude of pure epoxy resin and 0.05 wt% epoxy resin–GO composite materials were almost the same when the applied voltage was 16~24 kV, and the change trend was basically the same, as all slowly increased when the applied voltage increased. When the applied voltage was 28 kV, the maximum and average discharge amplitudes of pure epoxy resin were 184.2 mV and 100.6 mV, respectively. Furthermore, the maximum and average discharge amplitudes of 0.05 wt% epoxy resin–GO composites were 157.8 mV and 81.9 mV, respectively. Thereafter, the intensity of partial discharge of epoxy resin regulated by nano GO was weaker than that of pure epoxy resin. When the applied voltage was 32 kV and the critical flashover stage was reached, obvious through-discharge channels appeared on the surface of the sample, and the maximum and average discharge amplitudes sharply increased. The maximum and average discharge amplitudes of 0.05 wt% epoxy resin–GO composites also increased significantly, to 263.8 mV and 148.7 mV, respectively, i.e., still lower than that of pure epoxy. Figure 6 and Figure 7 demonstrates that although 0.05 wt% of the composite could inhibit the flashover voltage, the discharge amplitude during the discharge process was lower than that of the pure epoxy resin, especially in the near flashover stage. Moreover, the discharge amplitude of the pure epoxy resin was much higher than that of the 0.05 wt% composite.

**Figure 8:**
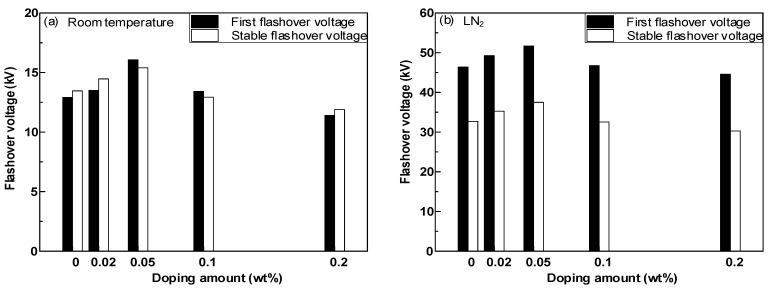
DC surface flashover voltage of epoxy resin–GO composites. (**a**) DC surface flashover voltage of epoxy resin–GO composites at room temperature; (**b**) DC surface flashover voltage of epoxy resin–GO composites at LN_2_.

Six flashover tests were carried out on each sample at room temperature and LN_2_ temperature, and their average values were taken as stable flashover voltage values. The first flashover voltage and stable flashover voltage of epoxy resin–GO composites with different doping mass fractions are shown in Figure 8a,b. The first flashover voltage and stable flashover voltage of composites at room temperature first increased and then decreased when the GO doping mass ratio increased, before peaking at 0.05 wt%. Under LN_2_ conditions, with an increase in the nano GO doping mass fraction, the first flashover voltage and stable flashover voltage of the composite material both increased first and then decreased. When the mass fraction of GO doping was 0.05 wt%, the first flashover voltage and stable flashover voltage reached the maximum values of 51.67 kV and 37.49 kV, respectively. When the mass fraction of GO doping reached 0.2 wt%, the first flashover voltage and stable flashover voltage were lower than those of pure epoxy resin.

### 3.3. Analysis of Trap Distribution Characteristics and Surface Discharge Mechanism of Epoxy Resin–GO Composites

The surface potential and trap characteristics of epoxy resin–GO composites were analyzed by the isothermal surface potential decay (ISPD) method. In this method, the needle grid electrode was used to corona charge the material surface. After the charging voltage was removed, the surface potentiometer was used to test the dissipation process of the deposited charge on the surface of the composite material, and the surface potential decay (SPD) process of the material was recorded. The charge leaving and entering the trap, as well as the migration of the material, can greatly affect the SPD process. Therefore, the energy level distribution of positive and negative polarity traps on the surface of the material can be calculated according to the above data to analyze the surface charge behavior in the process of surface discharge [21,22,23].

**Figure 9:**
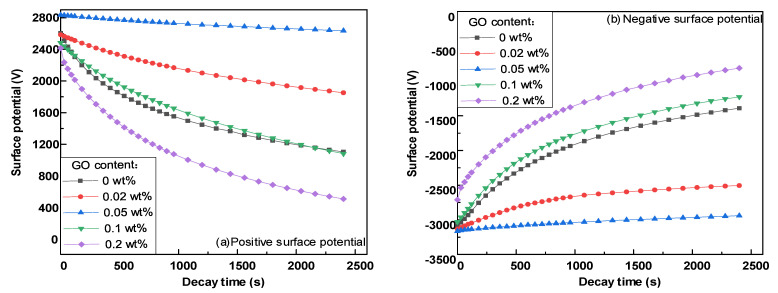
SPD characteristics of epoxy resin-GO composites.

**Figure 10:**
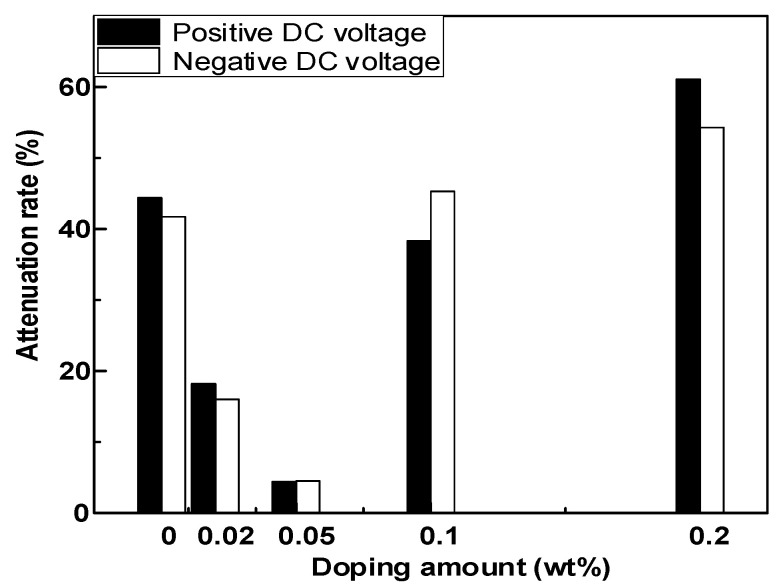
The 20 min surface potential attenuation rate of epoxy resin–GO composites.

Figure 9a,b show the surface potential attenuation characteristics of epoxy resin–GO composites under positive and negative corona, respectively. Figure 10 shows the surface potential decay rate data 20 min after charging. Under the condition of positive and negative corona, the surface potential attenuation trend of each sample was basically the same. With an increase in the nano GO doping mass fraction, the surface potential attenuation at 20 min first decreased sharply and then increased significantly. When the doping ratio was 0.02 wt%, the 20 min surface potential attenuation rates of positive and negative polarity were 18.2% and 16%, respectively. When the doping ratio was 0.05 wt%, it further decreased to the lowest values of 4.4% and 4.5%. As the doping ratio continued to increase, the 20 min surface potential decay rate increased rapidly. At 0.2 wt%, the 20 min surface potential decay rate was much higher than that of pure epoxy resin. The surface potential attenuation characteristics of epoxy resin–GO composites had a strong correlation with the doping mass ratio. When the doping mass ratio was low, the process of surface charge dissipation was significantly inhibited. When the doping quality was high, the dissipation process of surface charge was accelerated.

**Figure 11:**
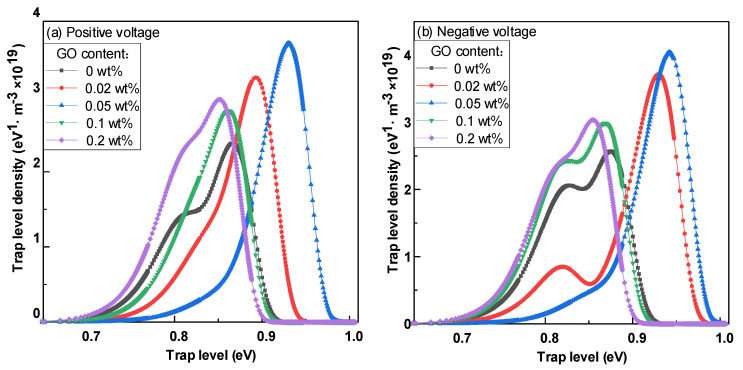
Surface trap distribution characteristics of epoxy resin-GO composites. (**a**) Surface trap distribution characteristics of epoxy resin-GO composites at positive voltage; (**b**) Surface trap distribution characteristics of epoxy resin-GO composites at negative voltage.

Figure 11a,b show the positive and negative trap distribution characteristics of epoxy resin–GO composites. With an increase in the nano GO doping ratio, the electron trap energy level and density and hole trap energy level and density showed an upward trend, and reached the extreme value at 0.05 wt%. When the doping ratio exceeded 0.05 wt%, for both electron and hole traps, the energy level and density decreased when the doping ratio increased, and the energy level distribution curve transitioned from an obvious double peak to a single peak. With the continuous increase in the doping ratio, the energy level and density of high-altitude hole and electron trap began to decrease, and the trap distribution curve demonstrated bimodal characteristics. Regardless of the doping ratio of the composite material, the energy level and density of the electron traps on the surface were higher than the hole traps. With an increase in the nano GO doping mass fraction, the hole and electron deep and shallow trap energy level centers of the composites basically increased first and then decreased. At the same time, the density of deep traps first increased and then decreased, while the density of shallow traps showed the opposite trend.

## 4. Discussion

The above tests show that the development process of surface discharge of epoxy resin in LN_2_ has step property, and because the flashover process in LN_2_ will not produce a large number of ionized gas molecules as in atmospheric environment, the variation law of flashover voltage is similar to that in room temperature atmospheric environment. Figure 3 shows that with an increase in the GO doping content, the surface conductivity of the composite first decreases and then increases. The surface conductivity of 0.05 wt% of the composite is the minimum in both a normal-temperature environment and a LN_2_ environment. Combined with the surface potential decay characteristics of the material in Figure 9, it can be seen that 0.05 wt% of the composite also has the slowest surface potential attenuation compared with other samples. The electrical properties of epoxy resin materials are improved by doping different contents of go materials, mainly by changing the surface trap energy level and deep and shallow trap density to regulate the surface conductivity, so as to improve the flashover voltage. The primary electron emission is the same as the flashover process under normal temperature atmospheric environment. It is emitted from the three junction points formed by the cathode electrode and its solid–liquid medium. However, due to the weak molecular thermal motion under a 77 K low-temperature environment, higher energy is required to produce effective primary electron emission, which leads to the surface discharge initiation voltage under LN_2_ condition, which is higher than that under a room temperature atmospheric environment, The number of primary electrons produced under the same conditions is much lower than that under room temperature air conditions.

At the same time, due to the high density of LN_2_ medium, most of the free travel of primary electrons is short, which is then absorbed and blocked by LN_2_, so it is difficult to produce secondary electrons. Only at the three binding points and the gas molecular layer adsorbed on the surface of the insulating medium can more effective secondary electron emission be generated. With an increase in the electric field intensity, partial discharge energy leads to LN_2_ gasification and bubbles appear on the material surface. At the same time, the adsorbed gas molecules on the material surface separate from the material surface, and the gasification channel continues to develop to the anode. Bubbles are also observed on the electrode and material surface in the test. A few primary electrons can obtain enough energy to impact the material surface or collide with gas molecules in the local gasification layer to produce secondary electrons, and the accompanying positive ions will settle on the material surface during movement due to low initial velocity, which intensifies the electric field distortion on the insulating material surface and the three junction points, resulting in more electron emission.

When enough collisions occur, a secondary electron avalanche and a certain partial discharge phenomenon are formed. The electron avalanche moves to the anode under the action of the electric field. At the same time, the positive charge accumulation on the surface of the insulating medium gradually intensifies, which intensifies the electric field distortion on the surface of the insulating medium and strengthens the secondary electron emission process. The pulse energy released by it will also aggravate the formation of the gasification layer on the material surface, and promote the scope of gasification channel to the anode area. When the discharge process develops to the critical flashover stage, the gasification channel between the Yin and Yang poles is formed. At this time, the electron has high energy and is less hindered. The surface discharge process will rapidly develop flashover and release energy in a very short time. During the experiment, a flash of high brightness and a large number of air bubbles are observed, and the amplitude of partial discharge signal increases significantly.

Therefore, based on the above experimental results, we believe that the surface discharge of epoxy resin in LN_2_ condition is related to flashover, the formation of desorbed gas on the material surface, and the formation of LN_2_ gasification layer. Once the gasification layer penetrates the positive and negative poles, the surface flashover breakdown will occur in a short time. Meanwhile, for the phenomenon that the first flashover voltage is obviously higher under the condition of LN_2_, it is considered that the higher voltage at the time of the first flashover and the higher energy released during the flashover can seriously destroy the material in the range of the flashover channel on the material surface and crack the surface structure of the material. At the same time, LN_2_ is vaporized to nitrogen and adsorbed on the surface of the material at high temperature, which causes the flashover voltage to drop greatly when subsequent flashover occurs. As the number of flashover increases, the surface damage of the material becomes more serious and the flashover voltage continues to decrease.

**Figure 12:**
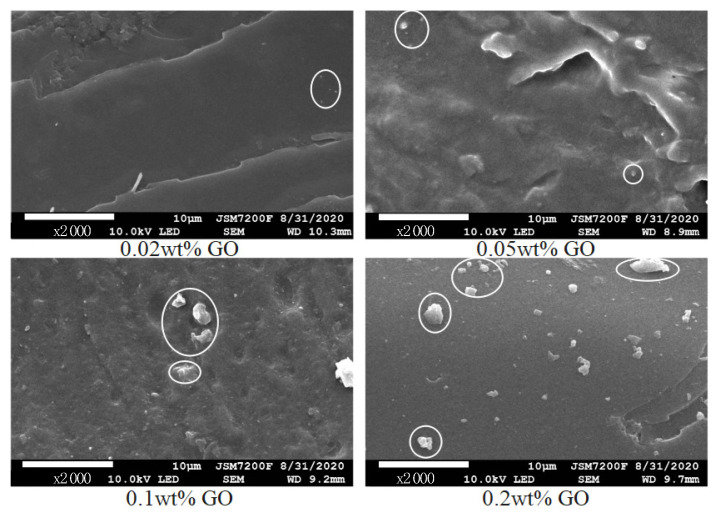
SEM images of epoxy resin–GO nanocomposites.

Figure 12 is the SEM diagram of cross-section morphology of composites with different go doping mass fraction. When the doping ratio is 0.02 wt% and 0.05 wt%, there is no obvious agglomeration in the composite, but with the continuous improvement in the doping mass fraction, some irregular aggregates begin to appear in the composite. However, the number of aggregates is small and the volume is relatively small. Relatively speaking, the doping of GO material is relatively uniform. After nano GO doping modification of epoxy composite insulation material, the trap energy level and deep trap density of the material will increase, which makes it difficult for electrons to escape the trap after being captured. At the same time, under LN_2_ conditions, the movement ability of electrons along the surface direction is weaker, which results in a large number of electrons being captured by the material surface during the movement process, thus inhibiting the initial electron emission and increasing the flashover voltage of the composite material. When the doping mass ratio of nanoparticles is too high, the trap energy level decreases and the density of the shallow trap increases. Thus, the electrons entering the trap can easy fall out under the action of electric field and reduce the flashover voltage.

For epoxy insulation materials, Al_2_O_3_ is also a common doping modification material; however, alumina is prone to large aggregates in the doping process, which will affect the insulation performance. Compared with Al_2_O_3_ and other doped epoxy materials, epoxy resin–GO nanocomposites can play a good regulatory effect under the condition of low proportion of doping. In addition, the phenomenon of agglomeration of materials is avoided in low proportion, and it will not have a great impact on other properties on the basis of improving the insulation performance. The effect of nano GO doping modification plays an important reference value for the design of superconducting power insulation.

## 5. Conclusions

High-temperature superconducting energy pipeline technology has the advantages of large capacity and zero resistance, which can simultaneously realize large-scale and long-distance transmission of power and LNG, and effectively reduce transmission loss. However, there is little research on the performance improvement of low-temperature insulating materials. The GO doping method is used to carry out the modification research of epoxy composite insulating materials for terminal use, and the surface discharge and flashover characteristics of composite insulating materials under room temperature and LN_2_ conditions are analyzed. The main conclusions are as follows.

(1) Due to the extremely low temperature of LN_2_ and its strong absorption and barrier effect, the maximum discharge amplitude and discharge repeatability of surface discharge of epoxy composite insulation remain low before critical flashover during the beginning and development of discharge compared with room temperature conditions. When the voltage reaches the critical flashover and the gasification channel is formed, the maximum discharge amplitude and discharge repeatability increase stepwise and rapidly.
(2) Under room temperature and LN_2_ conditions, the application of nano GO doping modification has an obvious effect on improving the electrical properties of epoxy composite insulation. Under room temperature and LN_2_ conditions, the flashover voltage of the composites first increases and then decreases, and reaches the highest when the GO doping ratio is 0.05 wt%. In addition, the flashover voltage at room temperature has no obvious relationship with the number of flashover, but the first flashover voltage of epoxy composite insulation under LN_2_ is significantly higher than that of subsequent flashover voltage.
(3) Under the condition of LN_2_, the development of surface discharge and the flashover process on epoxy composite insulating material are closely related to the formation of desorbed gas and the gasification layer on the material surface. Electron emission is suppressed and discharge develops slowly before the formation of a gas layer, penetrating the anode. Once the penetrating gasification layer is formed, the surface discharge can develop rapidly and flashover breakdown can quickly occur.

## Data Availability

All data are available in the main text.
